# Canonical pathways and selective mechanisms of autophagy in inflammatory bowel disease

**DOI:** 10.1007/s44307-026-00094-y

**Published:** 2026-02-13

**Authors:** Rui Lin, Zhaoyuan Xu, Min Zhi

**Affiliations:** 1 Department of Gastroenterology, The Sixth Affiliated Hospital, Sun Yat-sen University, Guangzhou, China; 2 Biomedical Innovation Center, The Sixth Affiliated Hospital, Sun Yat-sen University, Guangzhou, China; 3 Innovation Center of the Sixth Affiliated Hospital, School of Life Sciences of Sun Yat-sen University, Guangzhou, China

**Keywords:** Autophagy, Inflammatory bowel disease, Selective autophagy, Non-autophagic functions

## Abstract

Inflammatory bowel disease (IBD), encompassing Crohn’s disease (CD) and ulcerative colitis (UC), is a chronic inflammatory disorder of the gastrointestinal tract. Autophagy, an essential intracellular homeostatic process, plays a pivotal role in the pathogenesis and progression of IBD. This review systematically examines recent advances in understanding the involvement of autophagy in IBD, with a particular focus on the regulatory mechanisms governing its sequential phases—initiation, elongation, and termination—and their respective contributions to intestinal inflammation. We highlight how dysregulation of core autophagy components, including the ULK1 complex, Beclin 1 complex, and ATG16L1, influences inflammatory responses. Furthermore, this article delves into the context-dependent roles of selective autophagy pathways such as mitophagy, ER-phagy, and xenophagy in IBD, as well as the emerging significance of non-autophagic functions exerted by autophagy-related genes. By integrating these multifaceted aspects, this review aims to provide a theoretical foundation and identify potential targets for future precision therapeutics targeting autophagy in IBD.

## Introduction

Inflammatory bowel disease (IBD), comprising Crohn’s disease (CD) and ulcerative colitis (UC), is a chronic inflammatory condition of the gastrointestinal tract characterized by alternating periods of relapse and remission. CD may involve any segment of the gastrointestinal tract and commonly presents with fatigue, persistent or chronic diarrhea, abdominal pain, weight loss, and fever (Feuerstein and Cheifetz 2017). In contrast, UC is restricted to the colon and rectum, with typical symptoms including rectal bleeding, diarrhea, passage of mucus, and lower abdominal pain (Gros and Kaplan 2023). The extraintestinal manifestations of this disease are also very common and can affect multiple systems such as joints, skin, eyes, liver and gallbladder, lungs, pancreas, and blood vessels. Some manifestations (such as uveitis and PSC) even occur before intestinal symptoms (Gordon et al. 2024). Moreover, patients with IBD exhibit an elevated risk of developing other immune-mediated disorders, such as psoriasis, ankylosing spondylitis, and primary sclerosing cholangitis (Rogler et al. 2021).

In recent decades, the incidence of IBD has continued to rise, gradually shifting from a “rare disease” to a “less common disease.” Western countries exhibit a higher prevalence rate (Kaplan 2015). The sex- and age-standardized prevalence of IBD in the United States is 721 per 100,000 persons (95% CI 717–726), with UC prevalence at 378 per 100,000 (95% CI 375–382) and CD prevalence at 305 per 100,000 (95% CI 302–308) (Lewis et al. 2023). In China, both the incidence and prevalence of IBD have shown a consistent upward trend. The number of affected individuals increased from approximately 107,000 females and 133,000 males in 1990 to about 427,000 females and 484,000 males in 2019. Meanwhile, the age-standardized incidence rates rose from 1.20 per 100,000 to 2.65 per 100,000 in females and from 1.72 per 100,000 to 3.55 per 100,000 in males between 1990 and 2019 (Shao et al. 2022). In Japan, data from 2023 indicate that the number of patients with UC is approximately 316,900 (95% CI 223,900–409,900) and that of CD is about 95,700 (95% CI 61,100–130,400). Both figures represent a 1.4-fold increase over the eight-year period since 2015. The number of patients and the prevalence of both UC and CD in Japan have continued to show a steady upward trend (Tsutsui et al. 2025).

Although the precise etiology of IBD remains incompletely elucidated, several pivotal factors contributing to its pathogenesis have been identified. These can be broadly categorized into four domains: host immunity, intestinal microecology, environmental influences, and genetic susceptibility. Intestinal microbiota play a central role, and dysbiosis is known to trigger aberrant mucosal immune responses (Gilliland et al. 2024; Hill and Artis 2010). At the cellular level, intestinal homeostasis relies on dynamic interactions among intestinal epithelial cells (IECs), gut microbiota, and resident immune cells (Maloy and Powrie 2011). Accumulating evidence indicates that dysregulation of both innate and adaptive immunity underlies the aberrant intestinal inflammation observed in IBD. CD is primarily associated with Th1-driven responses, whereas UC is linked to atypical Th2 pathways (Gomez-Bris et al. 2023; Martini et al. 2023). Additionally, Th17 cells, which are a pro-inflammatory T-cell subset, expand in response to IL-23 stimulation (Geremia and Jewell 2012). Epidemiological studies further associate IBD with various environmental factors, including antibiotic use, microbial exposures during early and later life stages, and dietary patterns (Bager et al. 2012; Hviid et al. 2011; Piovani et al. 2019). Among genetic factors implicated in IBD, variations in autophagy-related genes are notably significant. Autophagy is an evolutionarily conserved process in eukaryotes involving lysosomal degradation of cytoplasmic components (Nguyen et al. 2013). Three primary forms of autophagy have been described: microautophagy, chaperone-mediated autophagy (CMA), and macroautophagy (Mizushima 2018). Initially characterized as a nonspecific bulk degradation mechanism induced by nutrient deprivation to recycle cellular components, autophagy is now recognized to also operate under basal conditions as a selective process targeting specific substrates such as protein aggregates, damaged mitochondria, and invasive microbes—a mechanism termed selective autophagy (Kim et al. 2019). Autophagy is integral to the maintenance of intestinal homeostasis, modulation of host–microbiota interactions, and coordination of innate and adaptive immune responses, as well as host defense against enteric pathogens (Mizushima 2018).

Although immune dysregulation and epithelial barrier defects are widely acknowledged as core mechanisms in IBD pathogenesis, existing research has predominantly focused on canonical autophagy pathways, particularly macroautophagy, in relation to IBD and inflammation. By contrast, the roles of selective autophagic pathways and the non-autophagic functions of autophagy-related genes in IBD remain inadequately systematized. This review therefore synthesizes recent evidence to systematically delineate the role of autophagy in IBD and its regulatory framework. It elaborates on the interplay between autophagy and IBD through three dimensions: canonical autophagy processes, selective autophagy, and non-autophagic functions of autophagy-related genes, thereby proposing novel targets and a theoretical foundation for precise therapeutic intervention.

## The relationship between canonical autophagy pathways and IBD

### Relationship between autophagy initiation and IBD

The initiation of autophagy and the subsequent formation of autophagosomes are coordinated by three core protein complexes. The process begins with the ULK1 kinase complex, comprising ULK1, ATG13, FIP200, and ATG101, which acts as the primary autophagy inducer (Ganley et al. 2009; Hosokawa et al. 2009; Mercer et al. 2009). Following induction, the ULK1 complex translocates to the initiation site and recruits the class III phosphatidylinositol 3-kinase (PI3K) complex, containing VPS34, Beclin 1, VPS15, and ATG14L. This complex generates phosphatidylinositol 3-phosphate (PI3P) on the phagophore membrane, serving as a platform for recruiting PI3P-binding effector proteins such as WIPI2 and DFCP1 (Zachari and Ganley 2017). The expanding phagophore then recruits the ATG12-ATG5-ATG16L1 complex, which functions similarly to an E3 ubiquitin ligase by promoting the lipidation of ATG8-family proteins (including LC3s and GABARAPs) with phosphatidylethanolamine. Lipidated ATG8 proteins embedded in the phagophore membrane facilitate cargo recognition, autophagosome closure, and eventual lysosomal fusion (Zachari and Ganley 2017).

ULK1 serves as a critical regulatory node in autophagy initiation, and its dysregulation is implicated in IBD pathogenesis. Upstream regulators of ULK1 include AMPK and mTOR, which exert context-dependent effects. AMPK, activated during energy stress in IECs, can either activate or inhibit ULK1, thereby modulating protective autophagy. For instance, under glucose deprivation, AMPK activation suppresses ULK1 and autophagy. However, during energy crisis, AMPK also protects ULK1 from caspase-mediated degradation, preserving cellular capacity to restore homeostasis upon stress relief (Park et al. 2023; Zachari and Ganley 2017). Conversely, mTOR, a kinase frequently activated in IBD by cytokines including TNF‑α and IL‑6, inhibits ULK1 and thus impairs autophagy, compromising the clearance of intracellular pathogens and damaged organelles (Zachari and Ganley 2017). Additionally, ULK1 activity is fine-tuned by ATG8 proteins: GABARAP and GABARAPL1 act as positive regulators, whereas LC3B and LC3C negatively regulate ULK1 (Grunwald et al. 2020).

ULK1 also regulates downstream autophagy components through phosphorylation. For example, ULK1 is palmitoylated by ZDHHC13, promoting its translocation to autophagosome formation sites and enhancing ATG14L phosphorylation, which is essential for VPS34 activation and PI3P production (Tabata et al. 2024). Emerging evidence indicates that ULK1 serves as a key regulator of intestinal homeostasis in IBD, exhibiting context-dependent pathogenic potential. Under conditions of nutrient deprivation or infectious stress, ULK1‑mediated phosphorylation of ATG16L1 at Ser278 is essential for efficient xenophagy and bacterial clearance (Alsaadi et al. 2019). Moreover, ULK1 activation promotes claudin‑2(CLDN2) degradation via AP2M1‑dependent endocytosis and autophagic flux, thereby enhancing intestinal barrier integrity. Given that CLDN2 is upregulated in active IBD and associated with diarrhea onset, defective ULK1 signaling may contribute to barrier disruption in genetically susceptible individuals (Ganapathy et al. 2021). CD patients commonly exhibit intestinal barrier impairment and bacterial translocation. If the ULK1‑ATG16L1 axis is functionally compromised, leading to diminished bacterial clearance, a persistent inflammatory response ensues. Mutations in ATG16L1 and single nucleotide polymorphisms (SNPs) in ULK1 are associated with CD (Henckaerts et al. 2011). However, in CD patients carrying the ATG16L1 T300A variant, the same phosphorylation event triggers caspase‑3‑mediated cleavage of ATG16L1, resulting in loss of function and more severe inflammation, which explains why T300A carriers are more susceptible to disease exacerbation under infection or stress (Alsaadi et al. 2019).

Beyond the canonical ULK1 complex, the scaffold protein AMBRA1 and the PI3KC3 complex (comprising VPS34 and UVRAG) play significant roles in intestinal epithelial homeostasis. AMBRA1 engages two non‑canonical pathways to regulate mucosal immunity: through its N‑terminal F1 domain, it competitively disrupts the binding of the PP4R1/PP4c phosphatase to IKKα/β, thereby sustaining NF‑κB activation (Xu et al. 2024). Meanwhile, another pathway antagonizes DUB3‑mediated deubiquitination of NRF2, accelerating proteasomal degradation of this antioxidant transcription factor and amplifying oxidative stress (Xu et al. 2025a, b). Recent studies have unveiled non‑canonical functions of PI3KC3 complex subunits in the intestinal epithelium. UVRAG, a target of miR‑125a, is regulated by the tiRNA‑Lys/EWSR1 axis and maintains barrier integrity by enhancing autophagic flux (Xu et al. 2025a, b). Conversely, DJ‑1 disrupts the VPS34‑Beclin1‑UVRAG complex through Rubicon degradation, thereby suppressing LAP‑dependent bacterial clearance (Gupta et al. 2022).

### Relationship between autophagy elongation and IBD

The elongation of the phagophore is critically regulated by the Beclin 1-VPS34 complex, which generates PI3P to recruit downstream factors necessary for membrane expansion. During elongation, cytosolic LC3 is cleaved by ATG4 to form LC3I, which is subsequently conjugated to phosphatidylethanolamine (PE) by the E1-like enzyme ATG7 and the E2-like enzyme ATG3, producing membrane-bound LC3-II. This lipidation process is facilitated by the ATG12-ATG5-ATG16L1 complex, which functions as an E3-like ligase. Phagophore expansion proceeds until the membrane seals to form a double-membraned autophagosome (Alula and Theiss 2023; Parzych and Klionsky 2014).

The abundance of LC3‑II (often represented by the LC3‑II/I ratio) is a widely used marker of autophagosomes. However, interpreting this ratio requires considerable caution. An elevated ratio may indicate enhanced autophagosome formation, but it can also reflect impaired lysosomal degradation downstream (i.e., blocked autophagic flux). Conversely, a decreased ratio, provided that autophagic flux is not obstructed, typically suggests reduced autophagosome synthesis. Therefore, definitive conclusions regarding autophagic activity depend on flux assays, such as the use of lysosomal inhibitors (e.g., bafilomycin A1) or tandem fluorescent LC3 reporter systems (Klionsky et al. 2012).

With this caveat in mind, studies reporting alterations in the LC3‑II/I ratio in IBD models offer clues to potential dysregulation during the elongation phase. A decrease in the LC3‑II/I ratio may suggest impaired autophagic elongation. At the cellular level, one study demonstrated that cGAS deficiency reduces Beclin‑1-VPS34 association, leading to attenuated LC3 lipidation and a decreased LC3‑II/I ratio. This elongation defect was accompanied by an increase in TUNEL^+^ epithelial cells, and forced restoration of elongation with rapamycin reversed the phenotype independently of STING–IFN signaling, indicating that the elongation step can be directly fine‑tuned by the pattern‑recognition receptor cGAS, beyond mere nutrient or mTOR sensing (Khan et al. 2022). Complementing these cellular findings, in vivo studies in IBD models have shown Il10^–/–^ mice exhibited a significant reduction in the LC3‑II/I ratio, which was restored by xylo‑oligosaccharide (XOS) intervention. The recovery of LC3‑II coincided with decreased bacterial penetration, restored goblet‑cell function, and down‑regulation of inflammatory factors, suggesting that functional recovery at the elongation phase may improve intestinal barrier function and suppress inflammation by enhancing autophagosome formation (Chen et al. 2022). Similarly, in an enteritis model, expressions of LC3‑II and Beclin‑1 were reduced, and a ketogenic diet (KD) restored their levels. Concurrent recovery of mitochondrial biogenesis markers (PGC‑1α, COX‑4) implied that the elongation phase may be linked to mitophagy. Impaired elongation could thus relate to defective mitochondrial clearance, subsequently affecting intestinal energy metabolism and inflammation, whereas restoration of elongation may improve intestinal cell function by promoting mitophagy (Chimienti et al. 2021).

Conversely, a marked increase in the LC3‑II/I ratio may indicate autophagosome accumulation, necessitating further investigation to determine whether it stems from enhanced initiation or blocked autophagic flux. At the cellular level, treatment with lactic acid bacteria (LAB)‑conditioned medium elevated the LC3‑II/I ratio in HCT116 cells and intestinal organoids, accompanied by up‑regulation of Beclin‑1 and ATG16L1. LAB‑induced elongation depended on vitamin D receptor (VDR) expression, as VDR‑deficient models showed no significant change in LC3‑II, indicating that probiotics regulate the autophagy elongation phase via the VDR signaling pathway (Lu et al. 2020). In several DSS‑induced animal models (Cheng et al. 2022; Dai et al. 2017; Ibrahim et al. 2025; Liu and Kong 2025), the LC3‑II/I ratio was consistently elevated. Intervention with the compound Chinese medicine Huangkui Lianchang Decoction (HLD) reduced the LC3‑II/I ratio and pro‑inflammatory cytokines (TNF‑α, IL‑6, IL‑1β) correlated positively with LC3‑II expression. While Beclin‑1 expression decreased, suggesting overall inhibition of autophagic flux (Cheng et al. 2022). Similarly, treatment with Jianpi Qingchang Decoction (JQD) lowered LC3‑II and Beclin‑1 expression. Moreover, autophagosome numbers in interstitial cells of Cajal (ICC) decreased, ICC structure recovered, and intestinal motility improved, implying that excessive activation during elongation may damage ICC and thereby impair intestinal motor function. Inhibiting the elongation phase could protect ICC and ameliorate IBD‑related functional symptoms such as diarrhea and tenesmus (Dai et al. 2017). Another study reported that overexpression of hsa_circ_0005255 significantly increased LC3‑II expression, upregulated Beclin‑1, and downregulated p62/SQSTM1. Mechanistically, hsa_circ_0005255 competitively binds miR‑23a‑3p, relieving its suppression of NCOA3 and thereby promoting the expression of elongation‑related proteins (Liu and Kong 2025). In the aforementioned studies, changes in Beclin‑1 protein paralleled those of LC3‑II, while mRNA levels remained unaltered, indicating that the elongation step is primarily regulated at the protein or lipidation level rather than transcriptionally. Clinically, LC3‑II levels varied across anatomical regions in the same CD patient: elevated in the mucosal layer but reduced in adipose tissue, with no difference in mRNA levels, suggesting post‑translational or lipidation‑based regulation. Impaired elongation in adipose tissue may weaken the capacity to clear bacteria or lipids (Leal et al. 2012).

The proper functioning of the autophagy elongation phase plays a crucial role in the overall autophagic process. When lysosomal degradation is not impaired, a decreased LC3‑II/I ratio indicates insufficient elongation, leading to hypo‑autophagy. This may contribute to intestinal barrier disruption and elevated levels of inflammatory factors in IBD patients. Conversely, a markedly elevated LC3‑II/I ratio can induce an excessive autophagic response, which similarly promotes increased inflammatory cytokines, intestinal motility disorders, and a weakened capacity to clear bacteria or lipids. Therefore, under conditions of unimpaired lysosomal degradation, a balanced LC3‑II/I ratio is essential for the normal progression of autophagy elongation. Both excessively high and low ratios can compromise the proper autophagic function in the intestinal tract.

### Relationship between autophagy termination and IBD

Autophagosome maturation is completed upon fusion with lysosomes, a process mediated by Rab GTPases, SNARE complexes, and the endosomal sorting complex required for transport (ESCRT). Following fusion, the resulting autolysosome acidifies and degrades the sequestered cargo, along with adapter proteins such as SQSTM1/p62. LC3-II is delipidated back to LC3-I, and the degradation products are released into the cytosol for reuse, thereby supporting cellular metabolism and survival (Sánchez-Martín and Komatsu 2018).

Crucially, evidence indicates that several IBD associated genetic variants do not intrinsically disrupt the terminal fusion step of autophagy. For example, cells harboring the CD-risk allele ATG16L1 T300A display normal LC3-II flux and SQSTM1 degradation during nutrient starvation, indicating intact autophagosome-lysosome fusion (Fujita et al. 2009). The pathophysiology may instead involve defective selective autophagy, particularly in Paneth cells. Similarly, IRGM deficiency impairs autolysosomal acidification and cargo degradation in response to adherent-invasive *E. coli* (AIEC) infection, a defect rescued by wild-type IRGM expression. This suggests that IRGM regulates lysosomal function rather than fusion machinery (Friswell et al. 2010). Furthermore, although the MTMR3 risk variant reduces PI3P levels and suppresses early autophagosome formation, the subsequent degradation of formed autophagosomes remains unaffected (Lahiri et al. 2015).

**Fig. 1:**
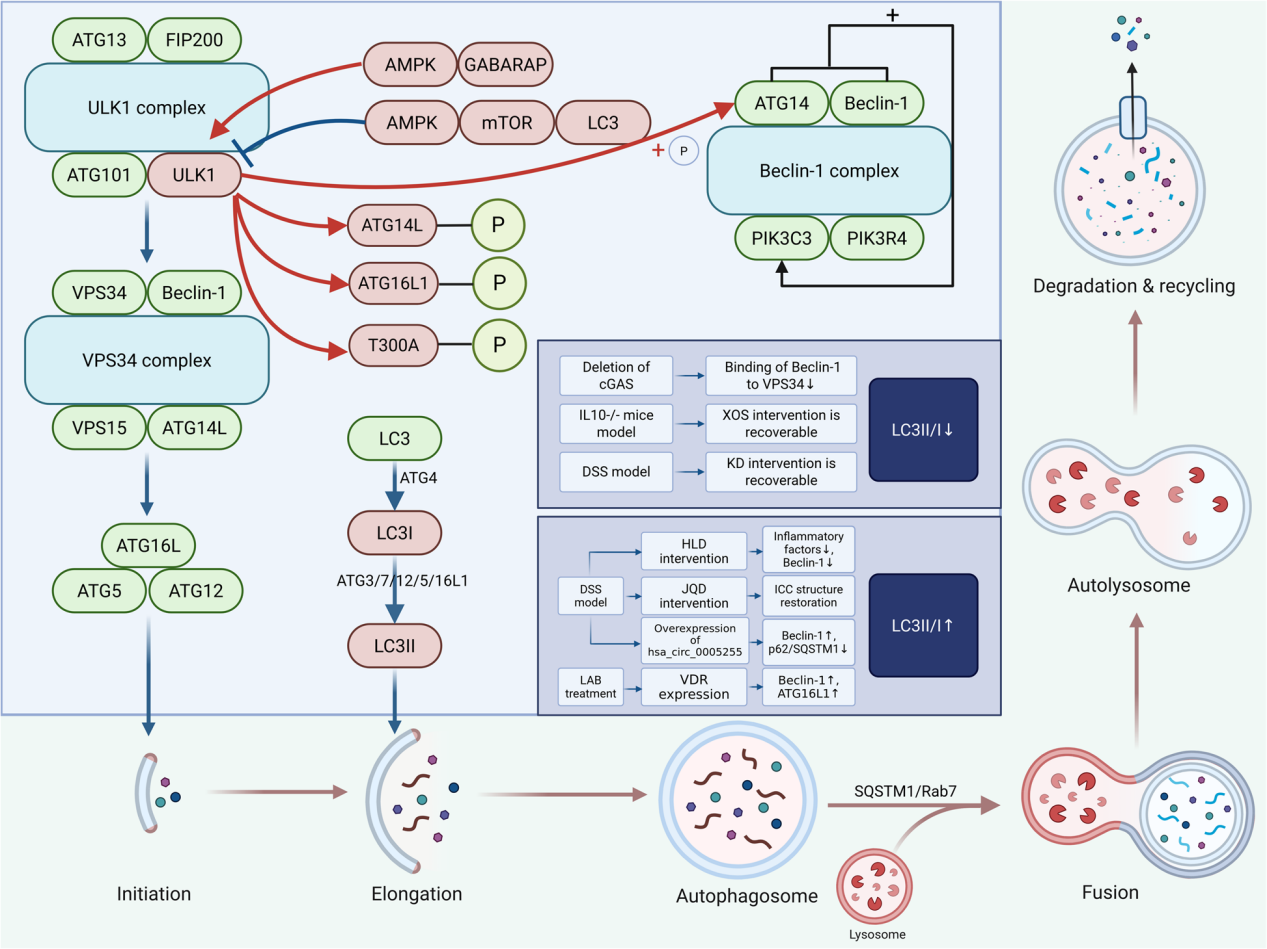
Canonical autophagy initiation-elongation-termination process and its regulatory factors. Abbreviations and Components ULK1 complex: ULK1, ATG13, FIP200, ATG101; VPS34 complex: VPS34, Beclin 1, VPS15, ATG14L; Beclin 1 complex: ATG14, Beclin 1, PIK3C3, PIK3R4; KD: Ketogenic diet; HLD: Huangkui lianchang decoction; JQD:Jianpi Qingchang decoction; VDR:Vitamin D receptor; LAB:Lactic acid bacteria; XOS: Xylo‑oligosaccharide

Collectively, these genetic studies indicate that impaired autophagic flux in IBD often originates in earlier, context-specific stages of autophagy. These defects may involve processes such as selective cargo recognition or lysosomal maturation, rather than representing a universal failure at the termination step (Fig. 1).

## The relationship between selective autophagy and IBD

### Overview of selective autophagy

Selective autophagy, a subtype of macroautophagy, mediates the targeted sequestration and degradation of specific intracellular components or organelles, including mitochondria, endoplasmic reticulum(ER), and invading microbes, in contrast to non-specific bulk degradation. Prominent forms include mitophagy, ER-phagy, and xenophagy, all of which have been implicated in the pathogenesis of intestinal inflammation. The following sections will discuss the roles of these three selective autophagy pathways in IBD.

### Mitophagy and IBD

Mitophagy is a selective autophagic process responsible for clearing damaged or dysfunctional mitochondria, thereby preventing the accumulation of ROS and maintaining cellular metabolic homeostasis (Zorov et al. 2014). This process is typically mediated by specific receptors such as Nix/BNIP3L or through the PINK1-Parkin pathway, which tag depolarized mitochondria for encapsulation by autophagosomes (Doblado et al. 2021; Ho and Theiss 2022; Zhang et al. 2021). Dysregulated mitophagy is associated with multiple pathologies, including neurodegenerative disorders, cancer, metabolic diseases, and IBD.

The regulatory impact of mitophagy appears to be cell type specific. In CD8^+ ^T cells, deletion of ATG7 or VPS34 increases mitochondrial mass, elevates ROS production, and promotes apoptosis (Arbogast and Gros 2018). Similarly, IRGM1 deficiency in CD8^+^ T cells leads to LC3B-II accumulation, reduced mitochondrial membrane potential, and enhanced glycolytic activity; inhibition of glycolysis with 2-deoxyglucose (2-DG) partially rescues T cell survival and function (Alwarawrah et al. 2022). Memory T cell persistence relies on fatty acid oxidation, which requires a healthy mitochondrial pool maintained by mitophagy. In contrast, CD4^+ ^T cells exhibit relative tolerance to mitophagy impairment, highlighting functional heterogeneity across lymphocyte subsets (Arbogast and Gros 2018). In germinal center B cells, WIPI2 deficiency disrupts mitophagy and impairs terminal differentiation, whereas loss of ATG5 or ATG16L1 does not significantly alter mitochondrial content, suggesting pathway-specific roles in B cell biology.

The clinical data show a connection between mitophagy and IBD. Low mitophagy function may lead to intensified intestinal inflammation, damage the intestinal barrier function, and cause apoptosis of intestinal epithelial cells. It was found in the ileal tissue of CD patients that the ATG16L1 T300A mutation caused low mitophagy function, resulting in reduced secretion of antimicrobial peptides in Paneth cells. In addition, LRRK2 can affect the initiation of mitophagy by phosphorylating proteins such as Rab29 and ULK1. Mutations in LRRK2 (such as G2019S) will disrupt mitochondrial homeostasis and cause CD (Alula and Theiss 2023). It was pointed out in a literature that mitochondrial damage-related molecular patterns (mtDNA, mtROS) can activate the NLRP3 inflammasome and promote the inflammatory cascade response. Mitophagy is a key mechanism for eliminating damaged mitochondria and inhibiting inflammatory responses, and it may become an intervention target in the 3PM (predictive—preventive—individualized) strategy (Arosa et al. 2024). Similarly, a significant decrease in the expression of ATF7 in the colonic mucosa was also observed in UC patients, and it was negatively correlated with the Mayo score. Mechanically, ATF7 promotes its transcription by directly binding to the PINK1 promoter, activates the Pink1/Parkin pathway, and initiates mitophagy. If ATF7 is absent, the expression of PINK1 decreases, leading to damage of mitophagy, causing an increase in ROS levels, an increase in IEC apoptosis, and an intensification of inflammation (Chen et al. 2025). Furthermore, a study pointed out that hair follicle mesenchymal stem cell exosomes (Hy-Exos) inhibit the PI3K/AKT/mTOR pathway through miR-214-3p, thereby restoring mitochondrial dynamics and improving the symptoms of DSS mice. Moreover, Hy-Exos can also restore Claudin1/Occludin to improve the colonic epithelial barrier, suggesting that exogenous activation of mitophagy can be used as a therapeutic strategy for IBD (Li et al. 2024). However, a study reported that in IBD, abnormally activated Piezo1 channels by mechanical forces cause an increase in Ca^2+^influx, leading to mitochondrial calcium overload, ROS outbreak, and ferroptosis caused by excessive mitophagy, thereby damaging the epithelial barrier (He et al. 2025). This suggests the role of bidirectional regulation of mitophagy in IBD. Therefore, normal mitophagy function is an important factor in maintaining intestinal homeostasis.

Parkin, a central effector in PINK1/Parkin-mediated mitophagy, also displays context-dependent functions. Several studies indicate that enhancing PINK1/Parkin signaling clears damaged mitochondria, suppresses ROS, and alleviates inflammation. In DSS-colitis, sodium butyrate (NaB) activates the Nrf2/HO-1 pathway to promote PINK1/Parkin-mediated mitophagy, thereby inhibiting NF-κB/NLRP3 activation and reducing inflammation (Bian et al. 2023). Similarly, myrtenol (MYR) and heat shock factor 2 (HSF2) enhance mitophagy through ANXA1 upregulation and PARL suppression, respectively (Li et al. 2025; Liang et al. 2022).

**Fig. 2:**
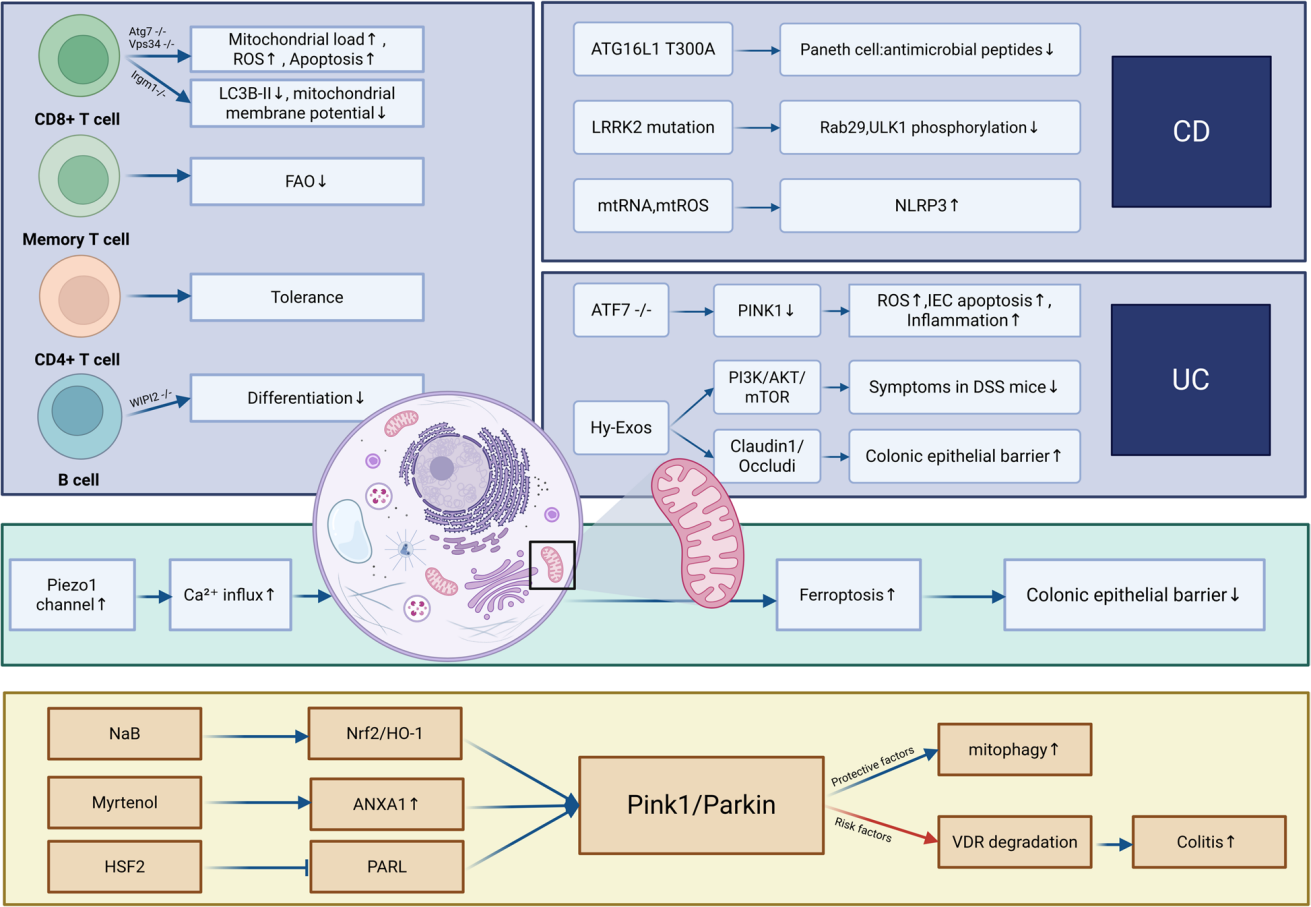
The relationship between mitophagy and IBD. Abbreviations Hy-Exos: hair follicle mesenchymal stem cell exosomes NaB: sodium butyrate HSF2: heat shock factor 2 IECs: intestinal epithelial cells

Paradoxically, Parkin can also exacerbate colitis by degrading non-mitochondrial substrates such as the vitamin D receptor (VDR). Parkin^−/−^ mice show reduced susceptibility to DSS-colitis, with attenuated weight loss, disease activity, inflammatory cytokines, and improved tight junction expression (Ma et al. 2023). These findings underscore the dual nature of Parkin in IBD (Fig. 2).

### ER-phagy and IBD

ER-phagy refers to the selective autophagic degradation of endoplasmic reticulum components. The ER, a dynamic network of tubules and cisternae, is essential for protein folding, lipid metabolism, and calcium storage. ER stress arises when protein-folding demand exceeds ER capacity, often triggered by inflammation, oxidative stress, or genetic mutations (Lin et al. 2008).

A bidirectional relationship exists between ER stress and autophagy, collectively influencing inflammatory outcomes in IBD. For instance, NOD2 and commensal microbiota alleviate ER stress by activating Beclin 1–dependent autophagy, facilitating clearance of misfolded proteins, enhancing mucin secretion from goblet cells, and reinforcing epithelial barrier function (Naama et al. 2023). Specialized secretory cells such as Paneth and goblet cells rely on the unfolded protein response (UPR) to manage high protein-folding loads. Moderate UPR activation supports the production of antimicrobial peptides and mucus, whereas sustained or severe ER stress shifts the response toward pro-apoptotic and pro-inflammatory signaling.

The IRE1α-XBP1 arm of the UPR exemplifies this duality: its activation under mild ER stress induces autophagy-related genes and facilitates clearance of dysfunctional ER, thereby resolving inflammation (Cao et al. 2024). In goblet cells, IRE1α nuclease activity enhances ER capacity and mucin output. Conversely, XBP1 deficiency leads to chronic ER stress, goblet cell apoptosis, mucus layer disruption, and worsened colitis (Heichler et al. 2020). Under persistent ER stress, the ATF6-CHOP axis inhibits autophagy by upregulating CSNK2B and HSPA5, downregulating Beclin 1 and LC3II, and promoting TNF-α and IL-6 release (Stengel et al. 2020b). ER stress also induces CLDN2 expression, which disrupts tight junctions; conversely, starvation-induced autophagy promotes CLDN2 degradation via AP2M1-LC3 interaction, thereby preserving barrier integrity (Ganapathy et al. 2021).

**Fig. 3:**
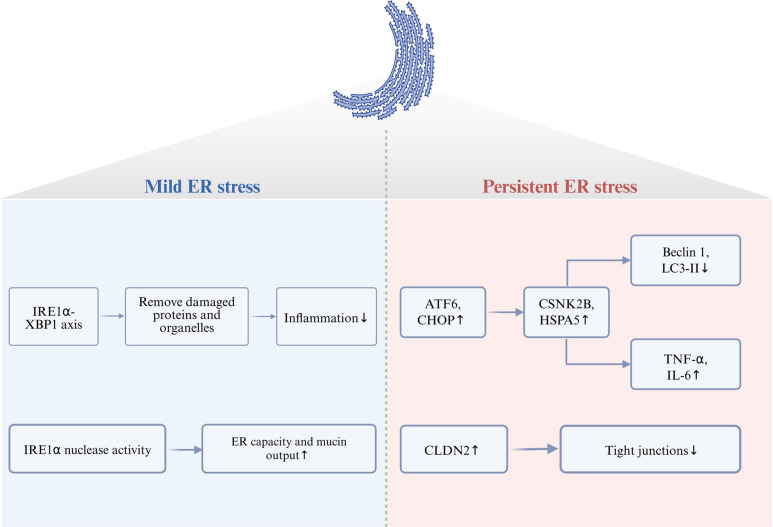
The relationship between ER stress and IBD

Clinical and experimental data further support the involvement of ER-phagy in IBD. Weighted gene co-expression network analysis (WGCNA) and immune infiltration studies have identified 11 ER stress–related genes (ERSRGs) that are upregulated in UC and correlate positively with neutrophil and M1 macrophage infiltration, as well as pro-inflammatory cytokine levels (Deng et al. 2023). These ERSRGs decrease following biologic therapy (e.g., infliximab, golimumab, ustekinumab), suggesting their potential as treatment response biomarkers. In enteroendocrine cells (EECs) of CD patients, ER stress-related genes (e.g., UBA5, PDIA3, CREB3L1) are upregulated (Kong et al. 2023). DSS-colitis models also show increased expression of UPR sensors IRE-1 and ATF-6 (Younes et al. 2024). Collectively, these findings position the ER stress–ER-phagy axis as a promising therapeutic target in IBD (Fig. 3).

### Xenophagy and IBD

Xenophagy is a specialized form of autophagy that involves the degradation and elimination of invading microorganisms such as bacteria, viruses, and parasites (Bauckman et al. 2015). During xenophagy, microorganisms are engulfed by autophagosomes and degraded in autophagosomes. Dysregulation of xenophagy is associated with a variety of infectious diseases, including tuberculosis, Salmonella infections, and viral infections such as influenza and HIV (Chen et al. 2023; Sharma et al. 2018).

In the context of IBD, xenophagy plays a critical role in clearing AIEC and Salmonella. Resveratrol promotes xenophagy by activating ULK1, NDP52, and p62, increasing LC3II levels, and enhancing bacterial clearance, accompanied by reduced TNF-α and IL-1β (Al Azzaz et al. 2018). Salmonella infection induces Golgi fragmentation, leading Paneth cells to employ a xenophagy-like mechanism involving LC3-positive vesicles to secrete lysozyme (Bel and Hooper 2018). Key regulatory factors include SACM1L, a PI4P phosphatase required for autophagosome-lysosome fusion; its loss causes PI4P accumulation and impairs bacterial clearance (Carey et al. 2022). Similarly, PI4K2A deficiency disrupts lysosomal PI4P pools, blocks autophagosome maturation, and leads to IBD-like symptoms (Dafsari et al. 2022). Pathway specificity is illustrated by RNF166, which is essential for clearing Shigella and Listeria but has minimal impact on Salmonella, indicating pathogen-selective regulation of xenophagy (Heath et al. 2016).

Genetic variations in xenophagy-related genes are strongly associated with IBD. The ATG16L1 T300A variant impairs AIEC clearance in macrophages and dendritic cells, leading to NLRP3 inflammasome hyperactivation, altered antigen presentation, and biased Th1/Th17 responses (Alula and Theiss 2023). Cell-specific effects are further demonstrated by the distinct outcomes of ATG16L1 deletion. In T cells, its loss skews immunity toward a Th2 phenotype and compromises antibacterial defense. Conversely, within B cells, xenophagy facilitates TLR9-dependent bacterial DNA sensing in effector subsets and contributes to the survival of memory B cells (Arbogast and Gros 2018).

IRGM mutations also disrupt xenophagy. A 20-kb deletion causes dose-dependent impairment in phagosome maturation, with homozygotes showing more severe defects than heterozygotes. Impaired bacterial clearance prolongs antigen exposure, leading to adaptive immune hyperactivation, ROS accumulation, genomic instability, and increased risk of colitis-associated cancer (Brest et al. 2010). A synonymous IRGM variant (c.313C > T) disrupts a miR-196 binding site, leading to aberrant IRGM expression and thereby demonstrating how non-coding regulatory mechanisms can influence xenophagy and IBD susceptibility (Brest et al. 2011).

**Fig. 4:**
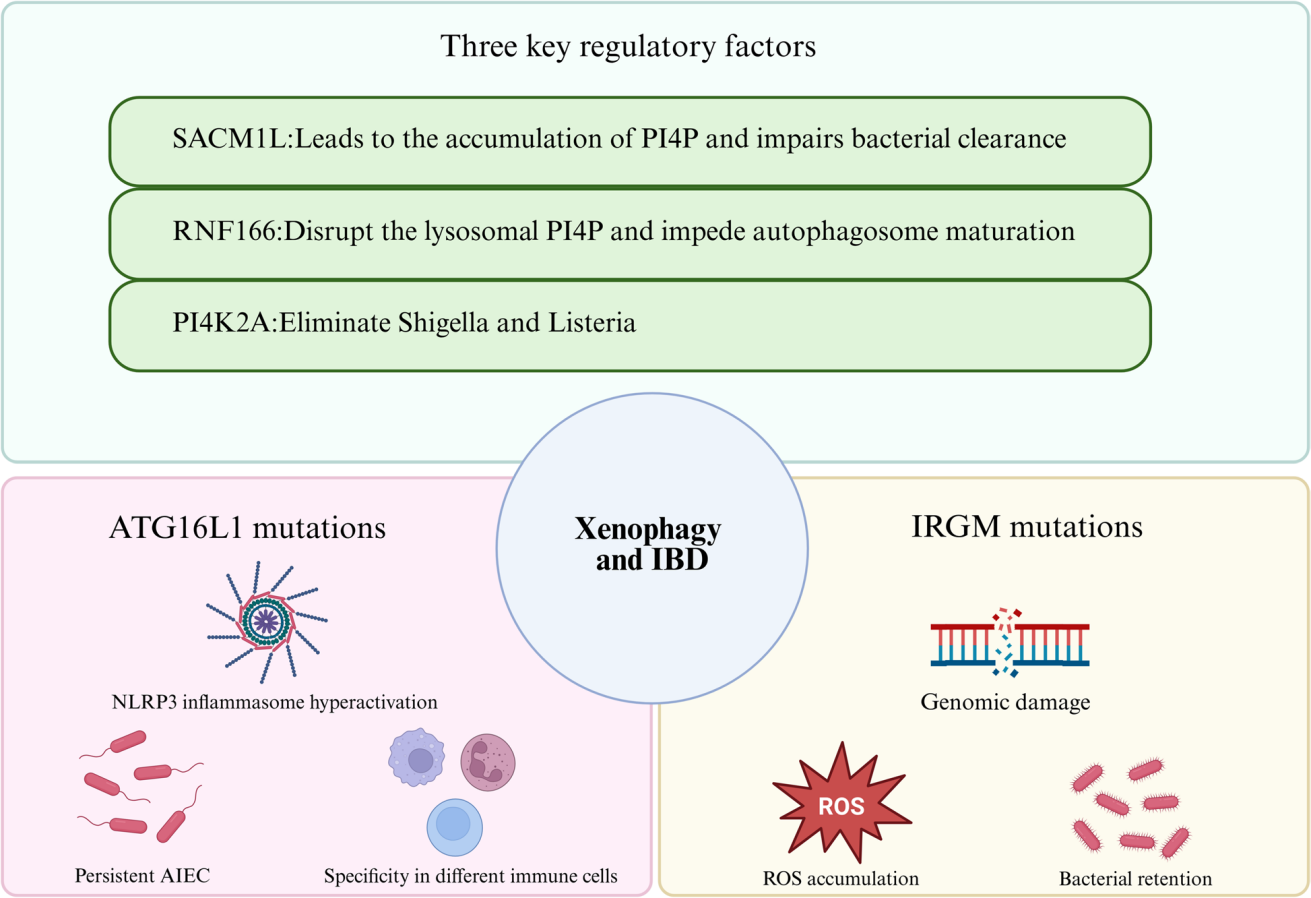
The relationship between xenophagy and IBD. Abbreviations AIEC:Adherent-invasive E. coli

Notably, xenophagy may exert dual effects depending on context. While it generally protects against intracellular pathogens, in Citrobacter rodentium infection, xenophagy deficiency paradoxically enhances immune activation and reduces bacterial load (Foerster et al. 2022). This suggests that xenophagy may be protective in acute infection but could suppress immune-mediated clearance or exacerbate tissue damage in chronic inflammatory settings. Pathogen type and infection stage are thus critical determinants of xenophagy’s role in IBD (Fig. 4).

## The relationship between non-autophagic functions of autophagy-related genes and IBD

**Fig. 5:**
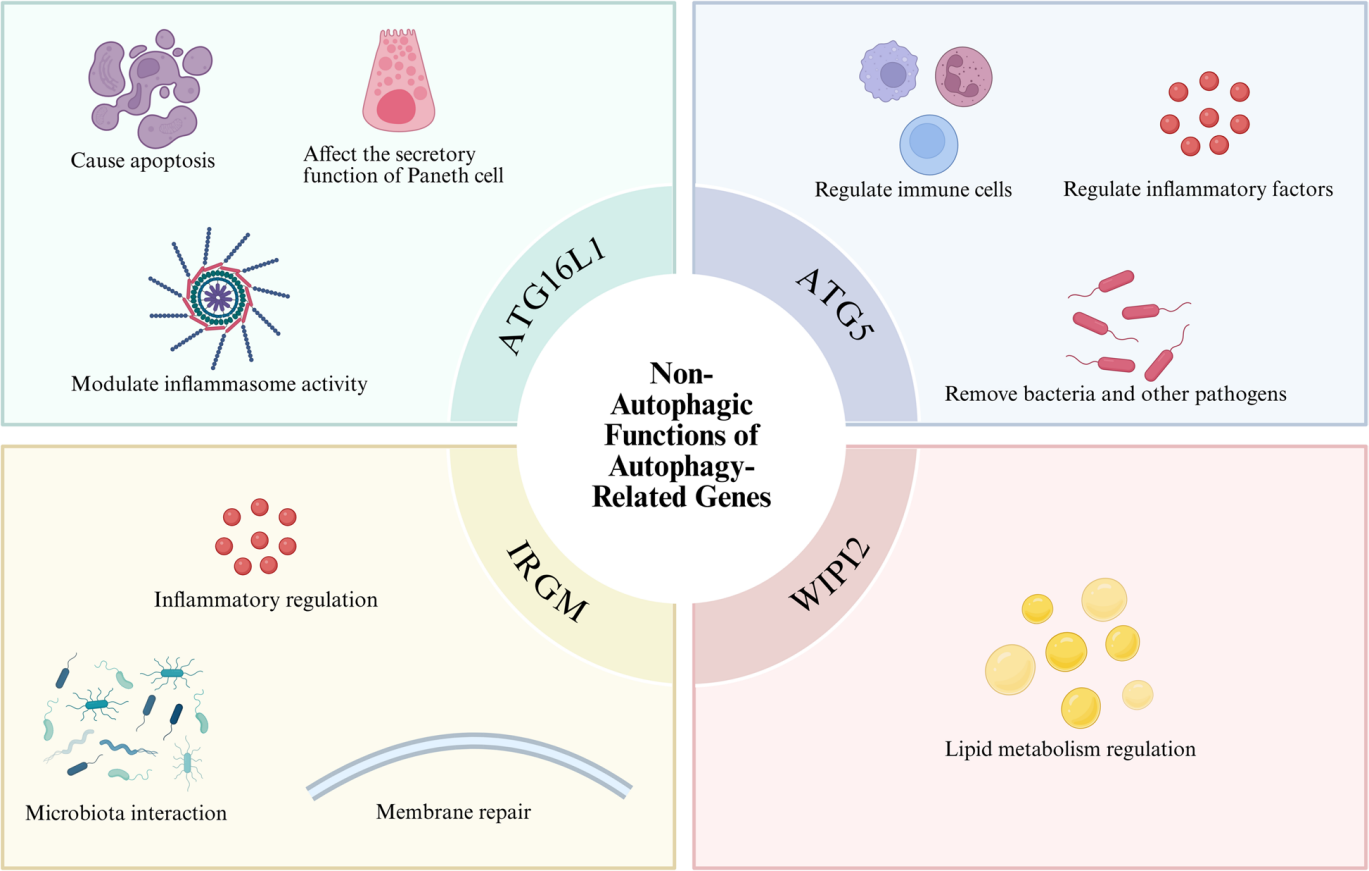
Non-autophagic functions of autophagy-related genes

As reviewed in previous sections, canonical autophagy pathways and selective autophagy are critically involved in IBD pathogenesis. Beyond these roles, a growing body of evidence indicates that autophagy-related genes may also contribute to disease through mechanisms that are not strictly dependent on autophagosome formation or lysosomal degradation. However, it should be noted that distinguishing between autophagic and non-autophagic functions is often challenging, as many autophagy-related proteins participate in multiple cellular pathways. This section summarizes the non-autophagic functions of key autophagy genes—ATG16L1, ATG5, IRGM, and WIPI2—and their implications in IBD (Fig. 5).

### ATG16L1

While ATG16L1 is well-established in autophagy initiation, elongation, and selective autophagy, there is increasing interest in its potential roles beyond autophagy in IBD. The common CD-associated variant ATG16L1 T300A has been proposed to enhance IEC sensitivity to IFN-γ–mediated apoptosis via the STAT1–caspase-8/3 axis, leading to Paneth cell loss and impaired epithelial repair—a mechanism that some studies suggest may operate independently of autophagy modulation, though this remains controversial (Foerster et al. 2023). In non‑epithelial contexts, the T300A variant has been linked to remodeling of the tumor immune microenvironment through mechanisms that may not require intact autophagic flux, such as promoting apoptosis and suppressing the EGFR/PPARγ pathway (Ma et al. 2021).

ATG16L1 also appears to regulate ER homeostasis in secretory cells. Its loss or mutation disrupts secretory granule architecture without impairing autophagosome formation (Adolph et al. 2013; Larabi et al. 2020). In Xbp1^ΔIEC^ mice, the T300A variant exacerbates ER stress and spontaneous ileitis, which is only partially alleviated by autophagy induction with sirolimus (Salem et al. 2015). Moreover, aged mice with epithelial‑specific ATG16L1 deletion develop ileitis even in the absence of XBP1 deficiency, further supporting possible autophagy‑independent contributions (Tschurtschenthaler et al. 2017). Notably, ATG16L1 deficiency does not block autophagosome formation but may amplify ATF6‑driven inflammatory signaling under ER stress (Stengel et al. 2020a).

Additionally, ATG16L1 T300A has been implicated in modulating inflammasome activity through mechanisms that some researchers argue are distinct from autophagy. It may impair assembly of the ATG16L1‑RIPK2 complex, leading to hyperactivation of NOD1/2 signaling and excessive IL‑1β/IL‑6 release (Sorbara et al. 2013). In ex-vivo human monocyte models, the T300A variant did not recapitulate autophagy defects seen in epithelial cells but selectively amplified IL-10/IL-6 responses (Glubb et al. 2011). Collectively, these findings suggest that ATG16L1 may influence epithelial apoptosis, Paneth cell function, and inflammation through pathways that could be partially independent of canonical autophagy, though the extent of this independence continues to be debated.

### ATG5

ATG5, a core autophagy protein, has also been reported to exert immunoregulatory functions beyond its role in autophagy. In B cells, ATG5 cooperates with LC3 and αv integrins to modulate TLR signaling, shifting the balance between NF-κB and IRF7 activation. ATG5-deficient B cells exhibit enhanced proliferation and antibody production upon TLR stimulation, a phenotype that appears independent of autophagic degradation but may be linked to altered TLR trafficking and signaling (Acharya et al. 2016). In thymic epithelial cells, ATG5 facilitates MHC-II-mediated antigen presentation and T cell selection; its loss disrupts T and B cell development (Bhattacharya and Eissa 2013), raising the possibility that ATG5 could similarly modulate intestinal immunity in IBD through both autophagic and non‑autophagic mechanisms.

ATG5 may also regulate cytokine production through pathways not strictly dependent on autophagy. It is highly expressed in lamina propria monocytes, where its upregulation correlates with increased IL-23 production (Ciccia et al. 2014). In addition, ATG5 deficiency has been associated with elevated secretion of inflammatory factors such as IL‑1β and IL‑18 (Bhattacharya & Eissa 2013). In IBD, ATG5 and ATG16L1 appear to jointly participate in regulating inflammatory responses, such as inhibiting the production of IL‑1β and IFN‑β (Hamaoui and Subtil 2022). These observations provide indirect support for the notion that ATG5 may help regulate inflammatory factors in IBD through mechanisms that could operate alongside or independently of autophagy, though definitive separation of these roles remains challenging.

Notably, autophagosome formation can still occur in ATG5‑deficient cells, and TRIM31‑mediated surrogate autophagy can clear Shigella even in the absence of ATG5. Conversely, TRIM31 knockout increases bacterial load despite normal ATG5 expression (Feng et al. 2022; Ra et al. 2016). In intestinal epithelial cells of CD patients, TRIM31 expression is downregulated by 45%, while ATG5 mRNA levels remain unchanged. This suggests that an adequate supply of ATG5 coupled with impairment of non‑autophagic compensatory pathways may be sufficient to drive IBD pathology, highlighting the complexity of disentangling autophagic from non‑autophagic functions. Overall, ATG5 may participate in regulating immune cells, controlling inflammatory factors, and eliminating pathogens through both canonical and non‑canonical pathways.

### IRGM

IRGM has been proposed to modulate inflammation, membrane repair, and host microbiota interactions through mechanisms that may not strictly require functional autophagy. It can directly bind the NLRP3 inflammasome and promotes its autophagic degradation in a ULK1/Beclin1-independent manner, relying instead on SQSTM1/p62 (Larabi et al. 2020). An IRGM GTPase mutant (S47N) that disrupts selective autophagy retains the ability to suppress inflammasome assembly, suggesting that IRGM’s immunoregulatory function might be separable from its role in autophagy, although the two pathways likely interact in vivo (Mehto et al. 2019). In DSS-colitis, IRGM deficiency exacerbates inflammation, an effect reversible with NLRP3 inhibition, underscoring its non-canonical role in limiting colitis.

In host microbiota interactions, IRGM scaffolds the NOD2-ATG16L1 complex at bacterial entry sites, promoting localized antibacterial responses without necessarily inducing full autophagic flux (Fritz et al. 2011). An ubiquitination-defective IRGM mutant (IRGM-KMUT) binds ATG16L1 but fails to restrict bacterial replication, indicating that IRGM’s antibacterial activity depends on ubiquitin-mediated signaling rather than autophagosome formation (Chauhan et al. 2016). In CD models, IRGM loss leads to intracellular bacterial accumulation (Fritz et al. 2011), highlighting the potential importance of its non-autophagic functions in microbial clearance.

IRGM also nucleates assembly of autophagy initiation complexes including ULK1 and Beclin1, without participating in later stages of autophagosome maturation (Chauhan et al. 2015). Human genetic studies reveal that IRGM-associated CD risk stems primarily from variations in promoter regions affecting expression, rather than coding changes affecting protein function (Massey and Parkes 2007), further supporting the physiological relevance of its non-autophagic roles though the interplay between transcriptional regulation and autophagic activity remains an area of active investigation.

### WIPI2

WIPI2 is primarily known for its role in autophagosome formation, but recent evidence suggests it may also participate in lipid metabolism regulation. Its function is not limited to autophagy initiation but may extend to mediating interactions between lipid droplets and autophagosomes. In the context of IBD, non‑alcoholic steatohepatitis (NASH) shares metabolic‑inflammatory pathways with IBD, raising the possibility that WIPI2 could influence IBD pathophysiology by modulating the lipid metabolism‑inflammatory axis, particularly in IBD subtypes associated with hepatic steatosis (Gui et al. 2019; Yu et al. 2023). However, whether these effects are truly independent of WIPI2’s autophagic functions remains unclear, and further studies are needed to dissect its potential non‑autophagic roles in intestinal inflammation.

## Dual regulatory role of autophagy in IBD

The role of autophagy in IBD is not unidirectional but exhibits a distinct duality. This section aims to systematically integrate the seemingly contradictory findings discussed earlier and provide an analytical framework explaining how autophagy can both maintain homeostasis and drive inflammation in IBD.

Across disease stages, autophagy typically plays a protective role during acute inflammation by clearing damaged organelles, invading pathogens, and misfolded proteins, thereby promoting cell survival and limiting inflammatory spread. For example, in DSS-induced acute colitis, probiotics or sodium butyrate alleviate mucosal damage by enhancing autophagic flux (Bian et al. 2023). However, in chronic or persistent inflammatory states, autophagic function may become hyperactivated, chronically suppressed, or undergo qualitative alteration, thereby transitioning into a pathogenic factor. Chronic LPS exposure can suppress autophagy elongation, leading to sustained inflammasome activation (François et al. 2014). Conversely, Parkin-mediated excessive mitophagy may, under certain conditions, trigger ferroptosis in epithelial cells (He et al. 2025). This suggests that moderate autophagy aids in restoring homeostasis, whereas sustained dysregulation results in energy exhaustion, cell death, and impaired tissue repair.

Across cell types, different cells exhibit varying demands and sensitivities to autophagy. Moderate autophagy, particularly selective autophagy, is crucial in IECs for maintaining barrier integrity and clearing endogenous damage (Alula and Theiss 2023). However, the ATG16L1 T300A variant promotes epithelial apoptosis via non‑autophagic pathways under IFN‑γ stimulation, highlighting the complexity by which genetic background and inflammatory microenvironment jointly determine the functional outcome of autophagy (Foerster et al. 2023). Paneth cells, as secretory cells, heavily rely on autophagy‑related proteins (e.g., ATG16L1, IRGM) to sustain granule secretion and antibacterial defense. Risk variants such as ATG16L1 T300A manifest as selective autophagy defects and granule abnormalities in Paneth cells, while basal autophagy may remain normal in other cell types (Adolph et al. 2013; Alula and Theiss 2023; Larabi et al. 2020). Furthermore, autophagy exhibits distinct regulatory patterns in T cells, B cells, and macrophages. For instance, in CD8⁺ T cells, autophagy maintains mitochondrial quality to support memory cell survival (Arbogast & Gros 2018). In macrophages, autophagy negatively regulates IL‑1β secretion by degrading the NLRP3 inflammasome. Consequently, deletion of the same autophagy gene may elicit opposing immune phenotypes across different immune cell subsets.

IBD‑associated autophagy gene risk variants (e.g., ATG16L1 T300A, IRGM) often reveal their pathogenic effects only in specific microenvironments. Under homeostatic conditions, cells carrying these variants may maintain normal autophagic flux. However, upon challenges such as inflammatory cytokines (e.g., TNF‑α, IFN‑γ), ER stress, or pathogen infection, their autophagic defects or non‑autophagic dysfunctions become amplified. For example, IRGM mutations lead to defective xenophagy and persistent bacterial survival in the context of AIEC infection, whereas they may show no obvious phenotype in a germ‑free setting (Brest et al. 2010, 2011). This underscores that the interaction between genetic risk and external environmental factors, particularly the gut microbiota, is key in determining the autophagic phenotype.

## Therapeutic prospects and targeted strategies

The therapeutic modulation of autophagy represents a promising frontier in IBD management. Current biological agents, including TNF-α inhibitors (e.g., infliximab, adalimumab), anti-integrin therapy (vedolizumab), IL-12/IL-23 antagonists (ustekinumab), and JAK inhibitors (upadacitinib), have been shown to exert their clinical effects, in part, through the regulation of autophagic pathways.

TNF-α inhibitors alleviate inflammation not only by neutralizing soluble TNF-α but also by restoring compromised autophagic clearance. In models of AIEC infection, anti-TNF-α treatment reduces bacterial load, a effect attributed to the recovery of xenophagic function (Azzman 2019). Mechanistically, infliximab enhances autophagic flux in macrophages, as evidenced by an increased LC3II/I ratio, decreased SQSTM1/p62 protein levels, and a greater number of LC3-positive puncta (Levin et al. 2016). Notably, the efficacy of anti- TNF-α therapy can be influenced by autophagy-related genetic variants. Patients carrying NOD2 or ATG16L1 risk alleles exhibit increased bacterial translocation, impaired neutrophil function, and elevated serum TNF-α, leading to accelerated drug clearance and reduced free drug levels. Consequently, these patients experience higher relapse rates within six months and often require intensified dosing regimens (Gutierrez et al. 2014).

Ustekinumab, a monoclonal antibody targeting the p40 subunit of IL-12 and IL-23, demonstrates efficacy in CD by modulating selective autophagy pathways. It reactivates autophagic flux and reduces mucosal calprotectin release primarily by inhibiting the IL-23/STAT3 signaling axis and diminishing SQSTM1/p62 dependent inflammasome activation (Ma et al. 2017). Within the microenvironment of perianal fistulas, ustekinumab mitigates oxidative stress by suppressing ULK1-mediated mitophagy, thereby reducing ROS accumulation and promoting fistula epithelialization (Chapuis-Biron et al. 2020). Intriguingly, unlike anti- TNF-α agents, ustekinumab’s effectiveness does not appear to be diminished in patients with the ATG16L1 T300A variant, suggesting its anti-inflammatory action may rely more on fine-tuning selective autophagy through the STAT3-p62 axis rather than on core macroautophagic machinery (Nuij et al. 2017).Small-molecule agents such as the JAK1 inhibitor upadacitinib also interface with autophagy. It alleviates ER stress and activates AMPK-driven protective autophagy through inhibition of the JAK/STAT pathway, contributing to its therapeutic benefit in IBD (Ahn et al. 2024).

Collectively, these findings elucidate a compelling mechanistic link whereby biological therapies alleviate IBD symptoms by positively modulating autophagic processes. This understanding not only provides a theoretical foundation for their use but also highlights autophagy as a key determinant of treatment response and a potential target for future personalized therapeutic strategies.

## Summary

Autophagy serves as a critical biological process intricately involved in the pathophysiology of IBD. Each phase of the canonical autophagy pathway, which comprises initiation, elongation, and termination, distinctly contributes to disease progression. Initiation is largely governed by the ULK1 complex and its upstream regulators; elongation depends on a tightly regulated LC3II/I ratio, where imbalance in either direction disrupts intestinal autophagic function; and termination also exerts specific regulatory influences in IBD.

Beyond canonical autophagy, selective autophagy pathways play specialized and often dual roles in intestinal homeostasis and inflammation. Mitophagy maintains mitochondrial quality, with Parkin serving as a key regulatory node whose function may exert both protective and detrimental effects. The interplay between ER stress and autophagy is bidirectional, collectively shaping inflammatory outcomes in IBD. Impaired xenophagy, frequently resulting from genetic variations, leads to intracellular bacterial persistence and exacerbated inflammation.

Finally, a growing body of evidence underscores the relevance of non-autophagic functions of autophagy-related proteins—including ATG16L1, ATG5, IRGM, and WIPI2—in modulating apoptosis, immune signaling, and metabolic pathways relevant to IBD. In addition, in Table 1, summarize the key autophagy-related genes and their associated genetic variations in IBD, the specific autophagy or non-autophagy defects they cause, and the resulting clinical phenotypes.

**Table 1:** Summary of key autophagy-related genes, variants, functional defects, and clinical phenotypes in IBD

Gene	Variant	Autophagic/Non-Autophagic Functional Defect	Specific Functional Impact	Clinical Phenotype/Association with IBD
ATG16L1	T300A	Autophagic Defect: Impairment in autophagosome formation, mitophagy, and xenophagy	• Reduced ability to clear intracellular bacteria (e.g., AIEC) • Decreased secretion of antimicrobial peptides from Paneth cells • Reduced stability of the ATG16L1 protein under stress conditions	Increased susceptibility to CD. Associated with ileal lesions, Paneth cell dysfunction, and sustained bacteria-driven inflammation
		Non-Autophagic Defect: Regulation of apoptosis, ER stress, and inflammasome activity	• Enhanced IFN-γ-induced apoptosis via the STAT1-caspase-8/3 axis • Exacerbated ER stress and inflammatory signaling (e.g., ATF6 pathway) • Impaired assembly of the ATG16L1-RIPK2 complex, leading to hyperactivation of NOD1/2 signaling and excessive release of IL-1β/IL-6	May contribute to epithelial barrier disruption, Paneth cell loss, and enhanced inflammatory responses independently of the canonical autophagy flux
ATG5	Deficiency	Autophagic Defect: Autophagy initiation	• Forms part of the third protein complex (ATG16L1-ATG5-ATG12) essential for autophagy initiation	Involved in immune dysregulation in IBD. Its deficiency may alter B/T cell responses, inflammatory cytokine profiles, and activate compensatory pathways such as TRIM31-mediated alternative autophagy
		Non-Autophagic Defect: Regulation of immune cells, modulation of inflammatory factors, and clearance of bacteria and other pathogens	• Modulates TLR signaling in B cells, balancing NF-κB and IRF7 pathways • Facilitates MHC-II-mediated antigen presentation in thymic epithelial cells • Regulates cytokine production, e.g., IL-23 in lamina propria monocytes	
IRGM	Mutation c.313C > T/Deletion	Autophagic Defect: Impairment in autophagy termination, mitophagy, and xenophagy	• Bacterial retention and hyperactivation of adaptive immunity • ROS accumulation and genomic instability • Observed in CD8^+^ T cells: accumulation of LC3B-II, decreased mitochondrial membrane potential, and enhanced glycolysis	May promote colitis-associated cancer
	GTPase activity mutant (S47N)	Non-Autophagic Defect: Inflammation regulation, membrane repair, and host-microbiota interactions	• Directly binds to the NLRP3 inflammasome, promoting its degradation in a manner independent of ULK1/Beclin1 • Scaffolds the NOD2–ATG16L1 complex at bacterial entry sites, initiating a localized antibacterial response that does not require full autophagic flux	Modulates inflammasome activity and coordinates early antibacterial defense
WIPI2	Deficiency	Autophagic Defect: Autophagy initiation and mitophagy	• Affects recruitment of downstream PI3P-binding proteins by the VPS34 complex • Impairs terminal differentiation of B cells	May play a role in IBD subtypes associated with metabolic dysfunction (e.g., fatty liver)
		Non-Autophagic Defect: Lipid metabolism	• May regulate lipid droplet metabolism and is implicated in the metabolism-inflammation axis	

By systematically synthesizing the roles of autophagy across its canonical, selective, and non-canonical dimensions, this review provides an integrated perspective on its involvement in IBD and highlights promising avenues for future mechanism-driven and autophagy-targeted therapeutic strategies.

## Data Availability

No datasets were generated or analyzed during the current study.
